# Investigating the Effects of Statins on Cellular Lipid Metabolism Using a Yeast Expression System

**DOI:** 10.1371/journal.pone.0008499

**Published:** 2009-12-30

**Authors:** Agata Leszczynska, Beata Burzynska, Danuta Plochocka, Joanna Kaminska, Magdalena Zimnicka, Magdalena Kania, Marek Kiliszek, Monika Wysocka-Kapcinska, Witold Danikiewicz, Anna Szkopinska

**Affiliations:** 1 Institute of Biochemistry and Biophysics PAS, Polish Academy of Sciences, Warsaw, Poland; 2 Institute of Organic Chemistry PAS, Polish Academy of Sciences, Warsaw, Poland; 3 Department of Cardiology, Medical University of Warsaw, Warsaw, Poland; University of Missouri, United States of America

## Abstract

In humans, defects in lipid metabolism are associated with a number of severe diseases such as atherosclerosis, obesity and type II diabetes. Hypercholesterolemia is a primary risk factor for coronary artery disease, the major cause of premature deaths in developed countries. Statins are inhibitors of 3-hydroxy-3-methylglutaryl-CoA reductase (HMGR), the key enzyme of the sterol synthesis pathway. Since yeast *Saccharomyces cerevisiae* harbours many counterparts of mammalian enzymes involved in lipid-synthesizing pathways, conclusions drawn from research with this single cell eukaryotic organism can be readily applied to higher eukaryotes. Using a yeast strain with deletions of both *HMG1* and *HMG2* genes (i.e. completely devoid of HMGR activity) with introduced wild-type or mutant form of human *HMGR* (*hHMGR*) gene we investigated the effects of statins on the lipid metabolism of the cell. The relative quantification of mRNA demonstrated a different effect of simvastatin on the expression of the wild-type and mutated *hHMGR* gene. GC/MS analyses showed a significant decrease of sterols and enhanced conversion of squalene and sterol precursors into ergosterol. This was accompanied by the mobilization of ergosterol precursors localized in lipid particles in the form of steryl esters visualized by confocal microscopy. Changes in the level of ergosterol and its precursors in cells treated with simvastatin depend on the mutation in the *hHMGR* gene. HPLC/MS analyses indicated a reduced level of phospholipids not connected with the mevalonic acid pathway. We detected two significant phenomena. First, cells treated with simvastatin develop an adaptive response compensating the lower activity of HMGR. This includes enhanced conversion of sterol precursors into ergosterol, mobilization of steryl esters and increased expression of the *hHMGR* gene. Second, statins cause a substantial drop in the level of glycerophospholipids.

## Introduction

Hypercholesterolemia is a primary risk factor for coronary artery disease, the major cause of premature death in developed countries. Lowering serum cholesterol levels has proved to be highly effective for cardiovascular risk reduction. Since cholesterol is synthesized by the mevalonate pathway, statins – inhibitors of the key enzyme of this pathway, 3-hydroxy-3-methylglutaryl coenzyme A reductase (HMGR), (P04035, P12683, P12684) – are now the most prescribed class of drugs worldwide [1], [2]. Statin therapy is regarded as well tolerated. Moreover, beside lowering the level of serum cholesterol, it has other positive effects such as those involved in improving the endothelial function, enhancing the stability of atherosclerotic plaques, decreasing oxidative stress and inflammation, and inhibiting the thrombogenic response [3]. However, serious adverse effects have been reported. Myopathy is of particular concern because of potential rhabdomyolysis. High-dose statin therapy may be hepatotoxic and may cause peripheral neuropathy, and it has also been postulated that it can promote tumor growth, particularly in women [4]–[7].

The high degree of conservation of the cellular lipid metabolism – from unicellular organisms to human cells – provides an excellent possibility to use yeast *Saccharomyces cerevisiae* for studying the general principles of these processes. Dissecting the pathways of lipid synthesis, storage and mobilization, as well as related regulatory processes, in the yeast model may contribute to the development of therapeutics and drugs against atherosclerosis, type II diabetes, obesity and other diseases related to dysfunctions of the lipid metabolism in humans.

In both yeast and humans the mevalonate pathway begins with the synthesis of 3-hydroxy-3-methylglutaryl-CoA (HMG-CoA) from acetyl-CoA. HMGR then converts HMG-CoA to mevalonate which is finally transformed into sterols. The human genome contains a single gene encoding an HMG-CoA reductase, whereas yeast (*S. cerevisiae*) cells contain two genes for this enzyme, designated *HMG1* and *HMG2*. Human and yeast reductases demonstrate functional and topological conservation. The sequences encoding the active site (COOH-terminal domain) of the human enzyme and the two yeast isozymes are very similar. Expression of the human HMG reductase in yeast cells restores viability of an *hmg1*Δ *hmg2*Δ double mutant, proving that the human reductase provides sufficient catalytic function to replace both yeast isozymes [8].

Research on statins concentrates mainly on the influence of statins on endogenous cholesterol biosynthesis, on the level of atherogenic low-density lipoproteins, and on the serum cholesterol level [1], [9]. Much less is known about the effects of statins on the lipid metabolism of individual cells. Additionally, data analyzing the impact of HMGR inhibition on the synthesis of lipids not connected with the mevalonate pathway, such as glycerophospholipids is scarce. In a previous study [10] we demonstrated that yeast may be used as a model to investigate the expression of wild-type and mutated forms of the human *HMGR* (*hHMGR*) gene. Moreover, the system proposed allowed us to investigate the effects of different statins and different forms of the hHMGR protein (wild-type or mutated) on the viability and growth rate of yeast cells.

In the study presented here, we investigated the impact of simvastatin treatment on the metabolism of sterols and glycerophospholipids within the cell, as well as on the expression of the *hHMGR* gene.

## Methods

### Ethics Statement

No, an ethics statement is not required for this work.

### Functional replacement of yeast HMG1 and HMG2 by wild-type and mutated forms of the hHMGR gene

The yeast strains Y06733 (MAT**a** his3Δ1 leu2Δ0 met15Δ0 ura3Δ hmg1::kanMX4) and Y16054 (MATα his3Δ1 leu2Δ0 lys2Δ0 ura3Δ hmg2::kanMX4) (EUROSCARF) were used in this study. The media as well as the genetic and microbiological techniques were essentially as in Rose et al. [11]. Simvastatin was extracted from Zocor® tablets (MERCK & CO, INC, Whitehouse Station, NJ, USA) by dissolving in pH 7.0 buffer containing 0.5% dodecyl sodium sulfate in 0.01 M sodium phosphate according to The United States Pharmacopoeia USP 26. The 10 mg/ml stock solution of simvastatin was stored at −20°C. Yeasts were cultured in synthetic media with or without 100 µM simvastatin for 24 h or 48 h.

The cDNA encoding *hHMGR* gene was amplified by PCR with the following primers: HMG-F 5′-TCTGGAGGATCCAAGGATTCTG and HMG-R 5′-ACCAAGTGGCTGTCTCAGTGAT, and was cloned into pGEM-T Easy (Promega, USA). The resulting plasmid was digested with *Bam*HI and *Eco*RI, and the fragment obtained was inserted into the *Bam*HI-*Eco*RI sites of the pUG36 yeast expression vector. The final plasmid encodes the wild-type *hHMGR* gene fused to an N-terminal yeGFP (yeast enhanced green fluorescent protein) tag, under the control of the yeast *MET25* promoter.

The overlap extension PCR according to Ho et al. [12] was used to introduce the point mutation: G→T (Q766H), into the *hHMGR* gene. This mutation was created to induce structural changes in the catalytic domain of the enzyme but without loss of its activity.

To construct a diploid strain heterozygous for the *hmg1*Δ and *hmg2*Δ mutations, transformation was performed with the pUG36 vector carrying the wild-type *hHMGR* gene, sporulated and dissected on complete or minimal medium. After tetrad analysis, a haploid spore clone was selected that was both kanamycin-resistant and auxotrophic for histidine – i.e. it carried both deletions. This strain, termed MB03-1D, required the plasmid-encoded *hHMGR* gene for viability.

### Determination of mRNA levels

Inocula of *S. cerevisiae* cells expressing the wild-type or mutated *hHMGR* gene were added to liquid minimal media supplemented with either simvastatin or buffer. The cells were grown with shaking at 30°C and collected after 24 and 48 h.

Total RNA was isolated from yeast cells using the RNeasy Mini Kit (QIAGEN, Germany). Reverse transcription (RT) was performed in duplicate using the QuantiTect Reverse Transcription Kit (QIAGEN, Germany), according to the manufacturer's recommendations. QPCR amplification was performed using a LightCycler 1.5 and the LightCycler FastStart DNA Master SYBR Green I (Roche Diagnostics GmbH, Germany) according to the manufacturer's instructions. The Pfaffl model [13] and the relative expression software tool (REST-384 ©) [14] were used to estimate the relative changes in mRNA levels of hHMGR in the analyzed strains cultured with or without simvastatin. Data normalization was carried out versus the transcript of the housekeeping 35S rRNA. The sequences of all primers and the qPCR amplification parameters are available upon request.

### Confocal microscopy

Cells were cultured overnight. The media were then supplemented with either 100 µM simvastatin or buffer and the cells were further grown with shaking for two hours at 30°C. To localize neutral lipids and glycerophospholipids in yeast cells, Nile Red staining was performed as described by Greenspan [15]. Briefly, 1.5 ml of cell culture were centrifuged at 200×g and resuspended in 500 µl PBS (150 mM NaCl and 20 mM sodium phosphate buffer, pH 8.0). To this PBS cell suspension, 5 µl of 1 mg/ml Nile Red solution (9-diethylamino-5H-benzo[α]phenoxazine-5-one, Sigma Chemical Co) in dimethyl sulphoxide (DMSO) were added and mixed. The cells were harvested by centrifugation at 200×g, washed twice with PBS and resuspended in 300 µl PBS for microscope analyses. They were plated on microscope glass slides and subjected to confocal laser scanning microscopy in the Laboratory of Confocal and Fluorescence Microscopy, Institute of Biochemistry and Biophysics, PAS. An EZ-C1 (Nikon) Eclipse TE2000-E microscope, equipped with a Plan Apo 60X oil objective (NA 1.4) was used. Excitation of neutral lipids was with an Argon-Ion laser at 488 nm, and emission was detected at 515/30 nm. Excitation of glycerophospholipids was at 543 nm, and emission was detected with a 610 LP filter. Images were collected with a EZ-C1 Confocal v. 3.6 program (Nikon) and processed with the EZ-C1 Viewer v. 3.6 (Nikon) and Adobe Photoshop 8.0.

### Lipid extraction

Yeast cells were harvested, washed with water, centrifuged, and their wet weight was estimated. Lipids were extracted according to the method of Folch [16] with some modifications. The cells were homogenized and suspended in chloroform ∶ methanol (2∶1) to a final volume equal to 20 times the volume of the cells. After dispersion, the mixture was agitated for 8 h at room temperature. The homogenate was centrifuged to recover the lipid phase. The organic solvent containing the lipids extracted was washed three times with 1/5 volume of 10 mM EDTA in 0.9% NaCl and evaporated to dryness in a nitrogen stream and suspended in hexane or benzene.

### Alkaline hydrolysis of lipids

Lipids extracted from yeast cells, suspended in benzene, were hydrolyzed at 95°C for 2 hours in a mixture of ethanol ∶ H_2_O (17∶3) containing 15% KOH (w/v). Lipophilic products were extracted with diethyl ether, washed with water, evaporated to dryness in a nitrogen stream, suspended in hexane and subjected to further analyses.

### TLC lipid analysis

Lipids were analyzed on Silica gel 60 F_254_ plates in the following solvent systems: A, chloroform ∶ methanol ∶ water (65∶25∶4); B, toluene ∶ ethyl acetate (95∶5). Glycerophospholipids were separated by two-dimensional chromatography, the 1st dimension being chloroform ∶ methanol ∶ 28% ammonium hydroxide (42∶18∶3), and the 2nd dimension chloroform ∶ methanol ∶ acetone ∶ acetic acid ∶ water (28∶12∶12∶6∶1). Lipids were visualized with iodine vapor.

### GC/MS analysis of sterols

GC/MS analysis of sterols was performed without derivatization on an HP6890N gas chromatograph (Agilent Technologies), coupled with a GCT Premier mass spectrometer (Waters/Micromass) equipped with a time-of-flight detector and an electron ionization ion source. An HP 5-MS column (30 m×0.25 mm, 0.25 µm) was used. Oven temperature was maintained at 150°C for 5 min, and was then increased linearly to 300°C at a rate of 5°C/min. The temperature of the injector and the transfer line was 250°C. Helium was used as a carrier gas at a flow rate of 1 ml/min in a constant flow mode. Mass spectra were acquired in a scan mode (1.2 sec per scan). Sterols were identified on the basis of their mass fragmentation patterns by comparing their spectra with those collected in the Wiley database (The Wiley Registry of Mass Spectral Data, 7th Edition, Wiley, 2000). In addition, the chemical ionization method was used to confirm the molecular mass of the sterols studied in one of the analyzed samples.

### HPLC/MS analysis of glycerophospholipids

HPLC/MS analysis was carried out using a High-Performance Liquid Chromatograph Prominence LC-20 (Shimadzu) coupled with a tandem mass spectrometer 4000 Q TRAP (Applied Biosystems Inc.). The mass spectrometer was equipped with an electrospray (ESI) ion source (Turbo Ion Spray) and a triple quadrupole/linear ion trap mass analyzer. HPLC separations were performed using a 4.6×150 mm Zorbax SIL RX (5 µm) column. Solvent A was hexane ∶ isopropanol (80∶20) and solvent B was isopropanol ∶ water ∶ triethylamine ∶ formic acid (88∶10∶0.6∶0.08). A linear gradient was used in all measurements. The flow rate was 0.5 ml/min. Mass spectra were acquired in the positive and negative ion mode. Nitrogen was used as the nebulizer and the curtain gas.

## Results

### Functional replacement of yeast HMG1 and HMG2 by wild-type and mutated forms of the human HMGR gene

cDNAs encoding the wild-type and mutant forms of *hHMGR* gene were expressed under the control of the yeast *MET25* promoter in a strain with deletions of both *HMG1* and *HMG2* (i.e. completely devoid of HMGR activity). The wild-type and Q766H forms of the enzyme restored viability of the yeast double deletion mutant, demonstrating that the human proteins – even the Q766H mutant in the catalytic domain – were catalytically active in yeast cells.

### Relative mRNA quantification of the hHMGR gene

Treatment of cells with simvastatin had a differentiating effect on the hHMGR mRNA level. After 24 h of growth, cells expressing the wild-type gene for hHMGR displayed a 30% increase in the relative mRNA level, while cells expressing a mutated form of this enzyme displayed only a slight increase in the mRNA level. Prolonged (48 h) treatment of cells with simvastatin caused an increase in the level of hHMGR mRNA by about 20% in cells expressing the mutated protein, whereas in cells expressing the wild-type protein the increase was about 50%. We observed similar effects of other statins on HMGR mRNA levels in cultures of HepG2 cells treated with various statins (results not shown).

### Lipids in wild-type yeast and in yeast expressing wild-type or mutated hHMGR gene

**Figure 1:**
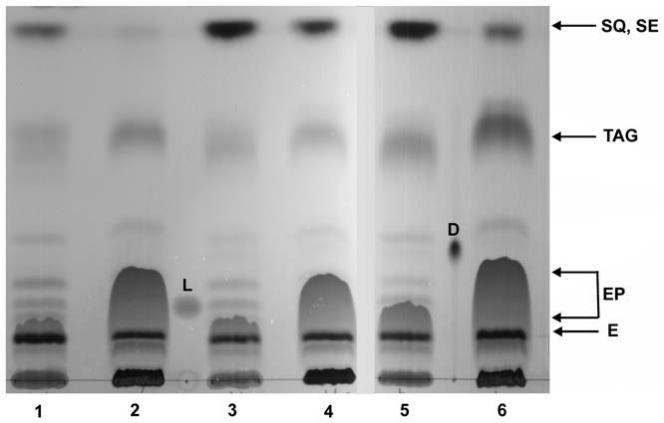
TLC analysis of simple lipids. A significant drop in the level of sterol precursors in cells cultivated in the presence of simvastatin indicates that the ergosterol intermediates were efficiently converted to ergosterol. The decrease of the steryl esters is also clearly visible after statin treatment. Lanes 1, 3, 5 lipids from cells harbouring the wild-type yeast, or the wild-type or mutated *hHMGR* gene, respectively. Lanes 2, 4, 6 lipids from cells treated with simvastatin harbouring the wild-type yeast, or the wild-type or mutated *hHMGR* gene, respectively. Abbreviations: SQ squalene, SE steryl esters, TAG triacylglycerols, EP ergosterol precursors, E ergosterol, L lanosterol, D dolichol composed of 16 isoprene residues.

To examine if changes in *hHMGR* gene expression level after simvastatin treatment influence the lipid metabolic pathways we analyzed the TLC separation patterns of lipids extracted from wild-type yeast cells and from cells expressing the wild-type or mutated form of *hHMGR* gene. As shown (Fig. 1 lanes 1, 3, 5) they are similar insofar as all strains contain steryl esters, squalene, triacylglycerols, and ergosterol and its precursors, though the quantities of particular classes of lipids vary. In all cells that were cultivated in the presence of simvastatin, we noticed a significant drop in the level of sterol precursors – they could not be detected on TLC plates in any strains (Fig. 1 lanes 2, 4, 6) indicating that the ergosterol intermediates were efficiently converted to ergosterol. The decrease of the steryl esters is also clearly visible after statin treatment (Fig. 1). The observation that ergosterol precursors were readily used to form ergosterol led us to investigate if statin treatment also induced the mobilization of sterols stored in the form of steryl esters in lipid particles.

### Confocal microscopy

**Figure 2:**
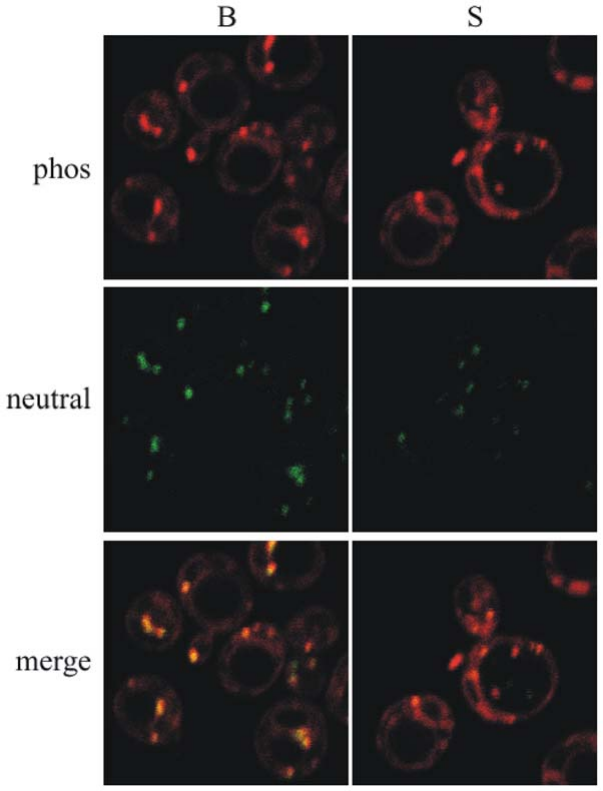
Nile Red staining of yeast lipid particles. Visualization of membranes composed of glycerophospholipids (red emission) and neutral lipids, triacyglycerols and steryl esters, in lipid particles (green emission). Cells were cultured overnight. The media were then supplemented with either 100 µM simvastatin or buffer and the cells were further grown with shaking for two hours at 30°C. To localize neutral lipids and glycerophospholipids in yeast cells, Nile Red staining was performed. Horizontal panels: upper glycrophospholipids at 543 nm excitation and 610 nm emission, middle neutral lipids at 488 nm excitation and 515/530 nm emission, lower merge of above panels. Vertical panels: B cells cultivated in buffer, S cells incubated for 2 h in buffer with simvastatin.

Lipid particles were observed applying confocal microscopy. Nile Red staining allows the visualization of membranes composed of glycerophospholipids (red emission) and neutral lipids, triacyglycerols and steryl esters, in lipid particles (green emission). As shown in Fig. 2 wild-type cells incubated with buffer had lipid particles containing neutral lipids that co-localized with phospholipids (yellow), whereas cells treated with simvastatin displayed a decrease in the intensity of both the green signal from steryl esters and the yellow signal from their co-localization with glycerophospholipids in lipid particles.

### Sterol precursor profiles for strains treated or not with simvastatin

**Figure 3:**
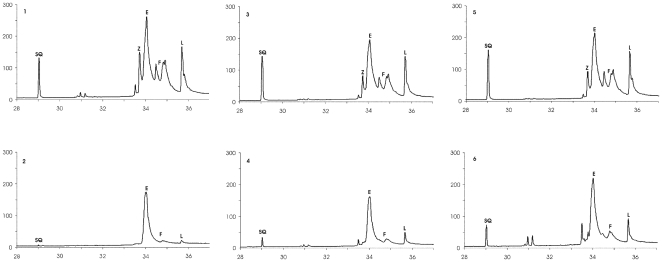
GC/MS analysis of sterols from yeast cells. Changes in the level of ergosterol and its precursors in cells treated with simvastatin depend on the mutation in the *hHMGR* gene. Panels 1, 3, 5 sterols from cells harbouring the wild-type yeast, or the wild-type or mutated *hHMGR* gene, respectively. Panels 2, 4, 6 sterols from cells treated with simvastatin harbouring the wild-type yeast, or the wild-type or mutated *hHMGR* gene, respectively. SQ squalene, Z zymosterol, E ergosterol, F fecosterol and isomers, L lanosterol and cholesta-8,14-dien 3-ol, 4.4-dimethyl (3∃, 5∀).

The results obtained during the analysis of lipids in strains treated or not with simvastatin prompted us to perform a more detailed analysis of ergosterol, sterol precursors and squalene present in cells harbouring wild-type and mutated forms of the *hHMGR* gene. Since steryl esters are the major source of ergosterol precursors, we performed their alkaline hydrolysis. GC/MS analyses revealed that in all the strains tested simvastatin treatment reduced the cellular sterol pool. Interestingly, the level of ergosterol was moderately lowered compared to that of total sterols whereas the amount of squalene was significantly decreased (Table 1). The strain harbouring the mutated form of the *hHMGR* gene displayed the smallest changes in sterol intermediates after statin treatment (Fig. 3).

**Table 1:** Decrease in sterols and squalene after simvastatin treatment.

Strain	Total sterols (%)	Ergosterol (%)	Squalene (%)
Wt	61	26	96
H	52	21	80
h	39	10	78

Lipids extracted from yeast cells were subjected to alkaline hydrolysis, purified and analysed by GC/MS.

Wt, wild-type yeast; H, yeast harbouring wild-type *hHMGR* gene; h, yeast harbouring the mutated *hHMGR* gene.

### Polar lipids in wild-type yeast and in yeast expressing the wild-type or mutated hHMGR gene

**Figure 4:**
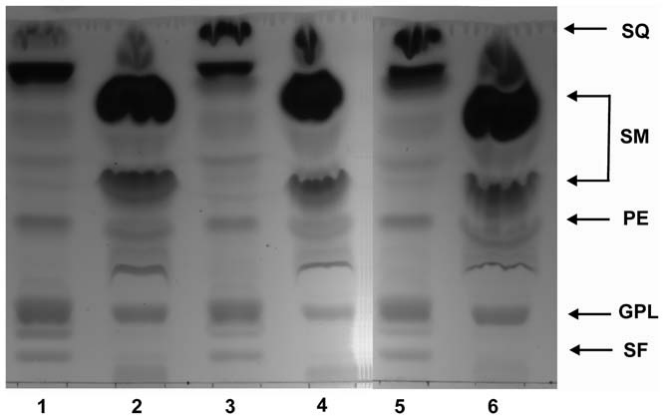
TLC analysis of complex lipids. Effect of simvastatin treatment on glycerophospholipids, apparently not connected with mevalonate pathway. Lanes 1, 3, 5 lipids from cells harbouring the wild-type yeast, or the wild-type or mutated *hHMGR* gene, respectively. Lanes 2, 4, 6 lipids from cells treated with simvastatin harbouring the wild-type yeast, or the wild-type or mutated *hHMGR* gene, respectively. Abbreviations: SQ squalene, SM simvastatin metabolites, PE phosphtidylethanolamine, GPL glycerophospholipids, SF sphingomyeline.

**Figure 5:**
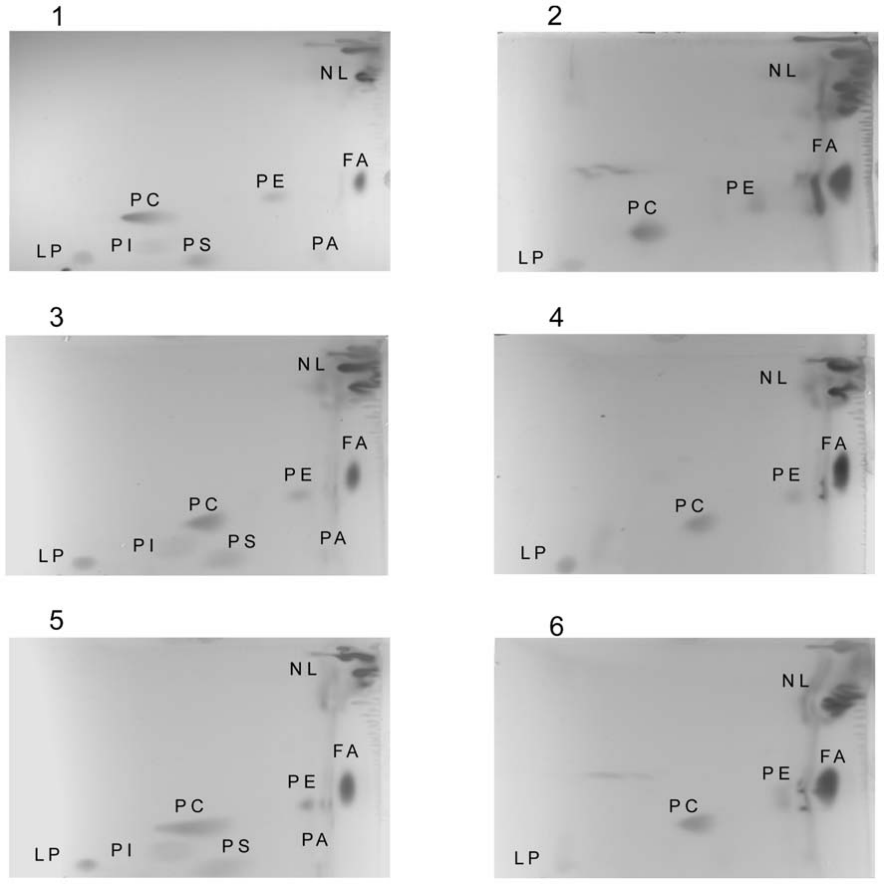
Two dimensional chromatography of glycerophospholipids. The levels of all major glycerophospholipids were diminished by treatment with simvastatin. Panels: 1, 3, 5 glycerophospholipids from cells harbouring the wild-type yeast, or the wild-type or mutated *hHMGR* gene, respectively. Panels 2, 4, 6 glycerophospholipids from simvastatin treated cells harbouring the wild-type yeast, or the wild-type or mutated *hHMGR* gene, respectively. Abbreviations: PC phosphtidylcholine, PE phosphtidylethanolamine, PS phosphatidylserine, PI phosphtidylinositol, PA phosphtidic acid, LP lysoglycerophospholipid, FA fatty acid, NL neutral lipids.

TLC analysis of polar lipids from all the yeast strains investigated showed that the patterns obtained are similar, independently of the type of HMG reductase the cells harbour (Fig. 4 lanes 1, 3, 5). Simvastatin treatment caused a decrease in the level of synthesis of lipids apparently not connected with the mevalonate pathway, such as glycerophospholipids (Fig. 4 lanes 2, 4, 6). Additional analyses, using two-dimensional chromatography and HPLC/MS for the identification of the compounds separated, demonstrated that the amounts of all major glycerophospholipids were diminished by treatment with simvastatin (Fig. 5).

## Discussion

Apart from their obvious hypocholesterolemic action, statins have been shown to exert many cholesterol-independent, pleiotropic effects, related to inflammation, oxidative stress, thrombosis and vascular function. However, molecular explanations for statin pleiotropy are generally scarce. Although the effect of statin treatment on the level of plasma cholesterol is well established, little is known about the impact of HMGR inhibitors on cell lipid metabolism.

Considering the fact that lipids are not only high-energy fuel storage molecules and building blocks of cell membranes but are also involved in modulating life processes in the cell, it is obvious that their homeostasis is of major importance. In previous studies we demonstrated that the yeast system is sensitive to statin treatment in terms of yeast cell viability and growth rate [10]. In the present study we observed that in yeast cells 24 h of simvastatin treatment induced expression of both wild-type and mutated *hHMGR* genes. Moreover, we demonstrated that the strength of this increase depends on the presence of a mutation in the *hHMGR* gene. This is in accordance with other studies, which showed increased expression of the *hHMGR* gene in HepG2 and L cells, as well as in human skeletal muscle-like cells following statin treatment [17], [18].

After treatment with simvastatin efficient conversion of precursors was observed, especially squalene, to ergosterol (Fig. 1, 3). Furthermore, within 2 h the signal intensity of steryl esters present in lipid particles, which are structurally reminiscent of mammalian lipoproteins particles [19] was significantly lowered suggesting that they were mobilized to compensate for the level of free sterols. Precursors are necessary for conversion to ergosterol that can directly serve as a component for membrane biogenesis and is the main constituent of the plasma membrane, similarly to cholesterol in mammalian cells [20]. Among the strains investigated the one with the mutated form of the *hHMGR* gene was the least sensitive to simvastatin treatment both in expression level of the mRNA of the *hHMGR* gene and in changes in the profile of sterol intermediates. This can be explained by poorer binding of the inhibitor to the active site of the mutated protein.

We postulate that inhibition of HMGR activity by statin treatment may trigger an adaptive response in the cell. First, the expression of the *hHMGR* gene is induced to compensate for the enzyme's inhibition. This finding is in accordance with the results of Gerber [17], who suggest that induction of the *hHMGR* gene compensates for the inhibition of the enzyme. Second, sterol precursors of ergosterol are efficiently used to maintain the physiological level of ergosterol.

Contrary to the effect of statin on plasma cholesterol level, knowledge about the influence of statin therapy on lipid fractions, such as phospholipids or sphingolipids is rather poor. Simvastatin was shown to decrease *de novo* synthesis of phosphatidylcholine via the cytidine diphosphate-choline pathway in HepG2 cell culture [21]. Another *in vitro* study proved that in combination with lipid restriction, atorvastatin reduced the phosphatidylserine pool by 50% [22]. Yet another study showed that pravastatin decreased plasma concentration of total phospholipids and LDL-phospholipids in hypercholesterolemic patients compared with the placebo group [23]. Vecka reported that fluvastatin treatment decreased the level of diphosphatidylglycerol in rat brain tissue [24]. The authors postulate that the alterations observed in brain lipid composition might be involved in genesis of neurological and mental symptoms following statin therapy. We demonstrate here that simvastatin treatment substantially decreased the amount of glycerophospholipids in all yeast strain tested.

Using the yeast expression system, two significant phenomena were detected. First, cells treated with simvastatin develop an adaptive response compensating for the lower level of sterols. This includes enhanced conversion of sterol precursors into ergosterol, mobilization of steryl esters and increased expression of the *HMGR* gene. Second, statins cause a substantial drop in the level of glycerophospholipids. To conclude, using the yeast expression system we demonstrated that statin treatment introduces significant changes in cell lipid metabolism.
